# Advantages of Ciprofol with Special Consideration of Pediatric Anesthesia

**DOI:** 10.3390/children13020188

**Published:** 2026-01-29

**Authors:** Alessandro Vittori, Cecilia Di Fabio, Marco Cascella, Franco Marinangeli, Elisa Francia, Ilaria Mascilini, Cecilia Maria Pizzo, Corrado Cecchetti, Valentina Di Conza, Teresa Grimaldi Capitello, Giuliano Marchetti, Giuseppe Servillo, Pasquale Buonanno

**Affiliations:** 1 Department of Anesthesia, Critical Care and Pain Medicine, ARCO, Ospedale Pediatrico Bambino Gesù IRCCS, Piazza S. Onofrio 4, 00165 Rome, Italy; 2 Department of Life, Health and Environmental Sciences (MeSVA), University of L’Aquila, Piazzale Salvatore Tommasi 1, Blocco 11, Coppito, 67010 L’Aquila, Italy; 3 Department of Medicine, Surgery and Dentistry, University of Salerno, Via Salvador Allende, 43, 84081 Baronissi, Italy; 4 Department of Neurosciences, Reproductive and Odontostomatological Sciences, University of Naples “Federico II”, Via Pansini, 5, 80131 Naples, Italy; 5 Clinical Psychology Unit, Department of Neuroscience, Ospedale Pediatrico Bambino Gesù IRCCS, Piazza S. Onofrio 4, 00165 Rome, Italy; 6 Surgery Unit, Bios Medical Center, Via Domenico Chelini 39, 00197 Rome, Italy

**Keywords:** ciprofol, cipepofol, HSK3486, anesthesia, pediatric anesthesia, pain, opioid, emergence delirium, children

## Abstract

The search for an ideal anesthetic has always been a major goal in anesthesiology. In recent years, the introduction of ciprofol has marked a major breakthrough in the pharmacological field, following the introduction of dexmedetomidine. Ciprofol has similar characteristics to propofol but with greater hemodynamic stability. Furthermore, it overcomes one of the most common discomforts associated with propofol: pain at the injection site. These characteristics make it a suitable hypnotic for pediatric use. Although studies on children are still limited, the literature on adults is now substantial and of high quality. The potential advantages of using ciprofol in pediatric anesthesia include pain-free induction, hemodynamic stability, less respiratory depression, and a lower incidence of emergence delirium.

## 1. Introduction

Over the past decades, propofol has been the cornerstone of intravenous anesthesia owing to its rapid onset of action, predictable pharmacokinetics, and swift elimination. However, its well-known limitations—including hypotension, respiratory depression, pain on injection, and the risk of propofol infusion syndrome (PRIS)—have motivated the search for alternatives with more favorable safety profiles [1,2,3].

**Figure 1:**
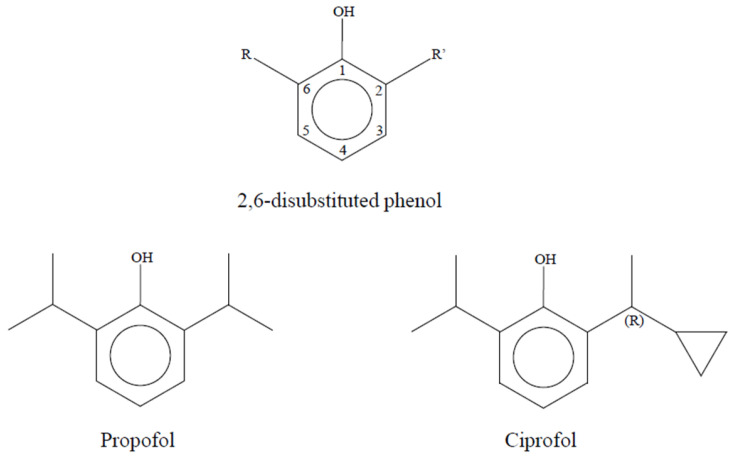
Chemical structure of propofol and ciprofol; both drugs derive from phenol with different substitution groups on carbon 2 and 6.

Ciprofol (HSK3486), also known as 2-(1-Cyclopropylethyl)-6-isopropylphenol, represents a new molecule belonging to the class of phenolic anesthetic derivatives (Figure 1). It was developed by the Haisco Pharmaceutical Group in China and was first reported in 2017 [4,5].

In recent years, numerous preclinical and clinical studies have confirmed that ciprofol provides anesthetic efficacy comparable to that of propofol but with greater hemodynamic stability and tolerability [6,7]. Ciprofol has been used in primary surgery, inpatients and outpatients, and also for sedation in intensive care settings.

**Figure 2:**
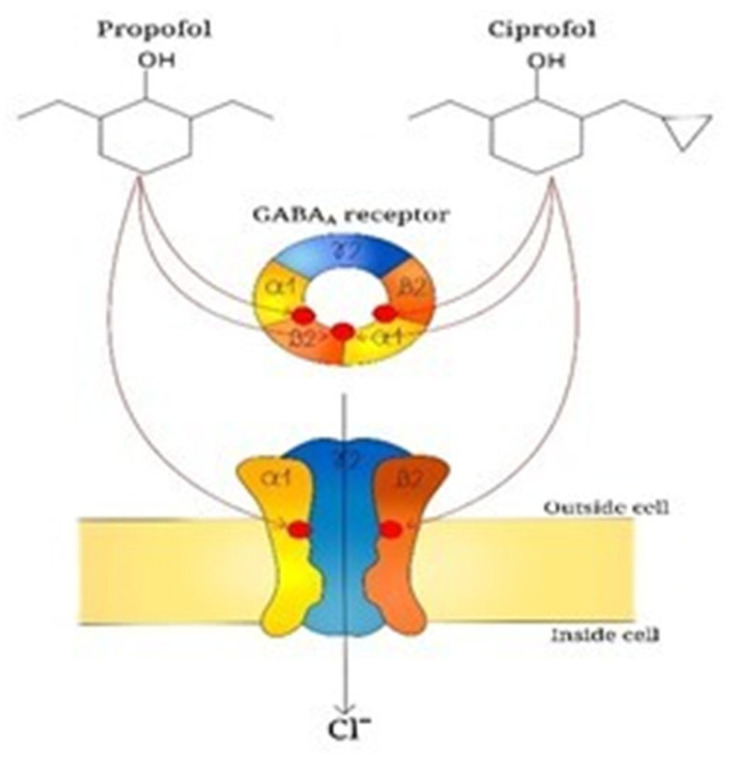
Propofol and ciprofol bind specific sites (red circles) between a1 and b2 subunits of GABA_A_ receptor; this interaction enhances the activity of GABA_A_ receptor which is an ion channel transporting chloride ions into the cells thus leading to a hyperpolarization of cell membrane and a suppression of exitability in the central nervous system.

The main sedative mechanism of ciprofol consists of positive allosteric modulation of GABA type A (GABAA) receptors. Competitive binding assays and whole-cell patch-clamp experiments have demonstrated that ciprofol induces chloride ion flux through competitive binding to bicyclophosphorothionate and t-butylbicycloorthobenzate sites on GABAA receptor channels (Figure 2) [8]. The activation of these chloride channels reduces the release of excitatory neurotransmitters such as glutamate, thereby producing sedation, hypnosis, and general anesthesia. It is a GABAA receptor agonist, with higher receptor affinity and an estimated potency approximately 4–5 times greater than that of propofol [9,10]. Its 10% lipid emulsion formulation allows continuous intravenous infusion with a reduced incidence of injection pain and a lower hemodynamic impact.

Ciprofol is a short-acting agent with rapid distribution in the central nervous system and equally fast elimination. After intravenous administration, bioavailability is complete, with an onset time of approximately 30–60 s and a mean duration of action of 5–10 min per single dose [8]. Its high solubility enables rapid penetration through the blood–brain barrier, followed by extensive distribution in adipose tissues. The volume of distribution is large, similar to that of propofol, but with a lower tendency to accumulate due to its more compact molecular structure [11].

Metabolism occurs primarily in the liver through oxidation, glucuronidation, and sulfation, resulting in the formation of the inactive metabolite M4-glucuronide, which is excreted renally. Ciprofol has a short elimination half-life, generally between 2 and 4 h, and is excreted mainly via the urine as inactive metabolites. Owing to its rapid metabolism and high clearance, the drug does not tend to accumulate in the body, even during prolonged infusions [12,13]

In animal models, ciprofol exhibits a broader LD_50_/ED_50_ (therapeutic index) compared to propofol (9.1 vs. 6.2), indicating greater dose-dependent safety [8]. LD_50_ is the dose at which a drug is lethal for 50% of animals tested, while ED_50_ is the dose of a drug that produces the intended pharmacological effect in 50% of the patient population studied during clinical trials [14,15].

Ciprofol possesses an anesthetic potency 4–6 times higher than that of propofol. This higher efficacy allows the use of lower doses, reducing hemodynamic and respiratory impact. In phase I studies, doses ranging from 0.3 to 0.5 mg/kg produced adequate sedation without significant adverse effects [16].

Numerous phase II and III clinical trials have evaluated ciprofol in digestive and respiratory endoscopy setting. In all cases, the agent demonstrated non-inferiority compared with propofol in terms of procedural success and sedation quality, but with greater hemodynamic stability and lower incidence of injection pain [16,17,18].

Ciprofol has also been evaluated as a sedative agent for mechanically ventilated patients, showing good manageability and safety for prolonged infusions [19]. Its short half-life allows fine modulation of sedation depth and rapid awakening upon infusion interruption—a crucial feature in intensive care management.

Ciprofol is generally well tolerated. The most commonly reported adverse events include mild hypotension, bradycardia, muscle fasciculations, and modest respiratory depression—all less severe than those observed with propofol [6,19,20].

Pain on injection, which occurs in up to 60% of propofol cases, is markedly reduced with ciprofol (<10%), owing to its lower concentration in the aqueous phase of the emulsion and lack of endothelial irritation [8].

To date, no cases of Propofol Infusion Syndrome (PRIS)-like syndrome have been reported in association with ciprofol, suggesting a more favorable metabolic safety profile [6].

Despite the current cost of ciprofol being approximately three times higher than that of propofol, large-scale industrial production is expected to reduce prices [8]. Improved hemodynamic stability, fewer adverse events, and shorter recovery times may offset the initial cost, enhancing overall cost-effectiveness.

These characteristics, combined with less burning at the injection site, make ciprofol an interesting hypnotic inducer in the field of pediatric anesthesia. Since most drugs used in pediatric anesthesia, as in other fields of medicine, often have to be adapted from those used in adult patients, the aim of this review is to highlight the characteristics of the drug that could make it useful and usable in the pediatric field as well. While studies conducted exclusively on children are few, the literature on adult patients is robust. Therefore, in this review, we will discuss the evidence that may support the use of ciprofol in children.

## 2. Research Methodology

A narrative literature review was conducted to evaluate the efficacy and safety of ciprofol in both pediatric and adult populations. Clinical and observational studies were identified through systematic searches of PubMed, Scopus, and the Cochrane Library. Articles published up to 2025 were considered.

## 3. Pharmacokinetics

Ciprofol and propofol belong to a class of compounds denominated 2,6-disubstituted phenols, which are characterized by a potent anesthetic activity. All these compounds share the capacity to bind and activate the γ-aminobutyric acid (GABA) receptor, thus inhibiting the synaptic transmission and the release of glutamate; the differences in terms of pharmacokinetic, pharmacodynamic properties, and the affinity to GABA receptors are strictly dependent on the steric bulk of substituting groups in position 2 and 6 of the phenol ring. Researchers focused their attention on ciprofol, mainly because of its higher potency with minimum structural changes and a modest decrease in hydrophilicity compared to propofol: in fact, effective dose 50 (ED_50_) of propofol and ciprofol in a validated rodent model of general anesthesia are 11.70 mg/kg and 1.5 mg/kg, respectively; lethal dose 50 (LD_50_) of propofol and ciprofol are 31.3 mg/kg and 9.9 mg/kg, respectively, with a dramatically more favorable therapeutic index (TI) for ciprofol (TI propofol vs. ciprofol: 2.7 vs. 6.6, respectively). Ciprofol differs from propofol by the substitution of a methyl group in one of the two lateral chains of the phenol ring with a cyclopropyl group: this change makes the molecular structure asymmetric and introduces a chiral center. R-stereoisomer of ciprofol showed to be more potent and with a higher affinity to the GABA_A_ receptor than S-stereoisomer and it represents the molecule currently traded and used in clinical practice. The higher potency of ciprofol allows for a decrease in the concentration of the drug in the whole emulsion and, more importantly, in the aqueous phase, which is the main cause of propofol injection pain. In animal studies, ciprofol preserved the rapid onset of propofol and showed a significant increase in the duration of action with a slight increase in recovery time, due to its tighter binding to the GABA receptor [4].

Hu C. et al. studied the pharmacokinetic and pharmacodynamic properties of ciprofol compared to propofol in healthy volunteers undergoing sedation through a continuous infusion of the drugs for 4 h (part 1 of the study) and 12 h (part 2); part 1 was characterized by an initial 30-min infusion of both drugs to reach the desired level of sedation, whereas in part 2 the induction was performed through a bolus, followed by a continuous infusion: the authors confirmed a lower incidence of injection pain with ciprofol and its higher potency, with consequent lower plasma concentration; no significant differences in half-life time, individual estimate of the terminal elimination rate constant, and mean residence time (i.e., the mean time the drug stays in the organism) were recorded. Volume of distribution (V_d_) and steady-state volume of distribution (V_ss_) of propofol and ciprofol were not significantly different during the 4 h infusion, whereas ciprofol showed a significantly lower V_d_ and V_ss_ during the 12 h infusion, which is likely due to its more lipophilic profile than propofol and the consequent capacity to leave plasma and enter the extravascular compartment [16].

In a single-center, single-arm, open-label, dose-escalation phase 1 clinical trial to evaluate the tolerability of a single intravenous injection of ciprofol emulsion, plasma peak concentration was achieved in 2 min for all the three doses tested (0.4–0.6–0.9 mg/kg); median plasma concentration at loss of verbal response was significantly higher in the group treated with 0.9 mg/kg (1750.0 ng/mL) than in the 0.4 and 0.6 mg/kg groups (809.5 ng/mL and 768.5 ng/mL, respectively); this variability could be attributed to the rapid distribution of ciprofol in central and peripheral tissue. Ciprofol plasma concentration for recovery of verbal response was similar in all the groups (about 300 ng/mL). C_max_, AUC_0–t_ and AUC_0–∞_ were not shown to be proportional to the dose, but this result should be affected by the small sample size of the study. Orientation recovery time varied from about 10 min in the 0.4 and 0.6 mg/kg groups to 18 min in the 0.9 mg/kg group. A single injection of ciprofol showed a dramatic difference in terminal half-life compared to continuous infusion (about 3 h vs. 11 h, respectively), and this factor should be carefully considered in clinical practice [16,21].

Ciprofol pharmacokinetic showed to follow a three-compartment model like propofol, while the pharmacokinetics of its main metabolite M4 (a glucuronide derivate) is best described by a two-compartment model. The estimated volumes of distribution and the clearances of the three compartments were 20.5 L, 176.0 L, and 61.7 L and 1.07 L/min, 1.27 L/min, and 3.27 L/min, respectively. BIS and MOAA/S followed a sigmoid Emax model and PD models were developed with a C_e50_ of 284 ng/mL and 326 ng/mL, respectively. BIS and the PK model were associated with a time delay at the effect-site concentration (Ce) (Te_lag_ = 0.455 min and 0.370 min, respectively) as opposed to the MOAA/S model which is not characterized by a time delay [21,22,23]. Early studies have shown that no dose adjustment is needed in patients with mild and moderate renal impairment. After injection of [^14^C]-labeled molecules, ciprofol was shown to be almost entirely transformed into M4, which is mainly excreted by the kidney (84.59%); only a minor part of ciprofol remained unchanged (3.97 %) and it was not found in urine; notably, the M4 metabolite does not show any hypnotic or toxic effect [23,24]. Cytochrome P450 (CYP) 2B6 and UDP-glucuronosyltransferase (UGT) 1A9 have been identified as the main enzymes attending ciprofol metabolism; notably, these enzymes are characterized by a high polymorphism, which could account for important interindividual differences in ciprofol’s pharmacokinetic and pharmacodynamic variability [25,26].

## 4. Pharmacodynamic

Teng Y et al. conduced a phase IIa open-label, non-randomized trial on patients undergoing colonoscopy to determine the best dosage of both ciprofol and propofol to use in a subsequent randomized, double-blind and propofol-controlled phase IIb study, which included 3 dosage groups treated with ciprofol 0.4 mg/kg, ciprofol 0.5 mg/kg, and propofol 2.0 mg/kg; they found that all the dosages were safe and well tolerated, reaching the 100% success rate for colonoscopy [27]. Anesthesiologists, patients, and endoscopists’ satisfaction rates were similar in the three groups with comparable recovery time and a total amount of the drugs administered that reflected the difference in the anesthetic potency of the two drugs observed in previous animal studies; the safety profile was similar in the three groups with mostly mild or moderate adverse effects. No differences between ciprofol and propofol were recorded in terms of respiratory and hemodynamic adverse effects; systolic pressure was characterized by a drop in the first 1–3 min and a subsequent recovering to values under the baseline; and patients in the ciprofol groups experienced less injection pain compared to the propofol group [27]. In a larger non-inferiority, randomized trial investigating patient sedation during colonoscopy and gastroscopy, authors found a similar induction time and comparable hemodynamic effects between the ciprofol and propofol groups; recovery time for ciprofol was longer only after colonoscopy procedures and this difference was attributed to the shorter duration of gastroscopy and the consequent need of fewer top-up doses to keep patients sedated: this finding suggests that the higher the dose of ciprofol, the longer the recovery time is, leading to an increasingly significant difference with propofol. Interestingly, ciprofol and propofol showed no significant differences in the hemodynamic parameters, but ciprofol was characterized by fewer respiratory complications such as respiratory depression, apnea, and hypoxemia [17]. These results were confirmed by the above-mentioned study of Hu et al. in healthy volunteers undergoing continuous infusion of ciprofol and propofol: injection pain was common in the propofol groups, no significant differences were reported in terms of safety and tolerability between ciprofol and propofol, and both drugs determined a decrease in blood pressure for the duration of the infusion [16]. Pharmacodynamic parameters such as the onset of sedation, recovery time and the trend of the bispectral index and Richmond Agitation–Sedation Scale were comparable between ciprofol and propofol infusion, with a slightly lower incidence of respiratory depression in patients treated with ciprofol. A ciprofol bolus dose as well as propofol can induce apnea and the prolonged infusion was associated with a higher incidence of adverse effects which, nevertheless, did not require any intervention [16]. Teng et al. also found that most of the AE (41 in 18 subjects, 83.3% of population) were drug-unrelated and mild; in particular, the adverse effects likely related to ciprofol administration were abnormal limb movement, sinus bradycardia, and prolonged QTcF; body movements were dose-dependent [27]. A slight drop (<10% of baseline) in arterial pressure occurred for 5 min after injection, becoming stable thereafter. The duration of loss of verbal response (LOR_verbal_), its recovery, the unresponsiveness to painful stimuli, the duration of BIS < 60, and the recovery of complete alertness were dose-dependent in the range of 0.4–0.9 mg/kg; in particular, the duration of BIS < 60 was similar to the duration of LOR_verbal_ and to the duration of MOAA/S ≤ 1 for every dosage tested [21].

Wang X et al. compared the use of ciprofol for the induction of general anesthesia and they found that it was not inferior to propofol in terms of induction success with a comparable incidence of adverse events; in particular, ciprofol 0.4 mg/kg showed a statistically significant longer time to successful induction and time to loss of eyelash reflex compared to propofol 2 mg/kg, even if this difference does not seem clinically relevant [28]. BIS values showed the same pattern for both drugs, with a rapid decrease after injection and a minimum at 4 min; notably, the variability of BIS values in the ciprofol group was much lower than in the propofol group, with a higher percentage of patients in the ciprofol group maintaining a BIS < 60 in the following 30 min after drug administration; moreover, even the number of patients experiencing an arterial pressure increase after drug administration was significantly lower than in the propofol group, thus indicating that ciprofol ensured a more stable sedation. Other studies in gynecological surgery confirmed the non-inferiority of ciprofol compared to propofol in anesthesia induction, with fewer intubation responses in the ciprofol group [12,28].

Ciprofol was also investigated for the induction and maintenance of general anesthesia during kidney transplantation: compared to propofol, ciprofol showed a faster loss of eyelash reflex and a shorter time to BIS < 60, with a prolonged recovery time and a smaller amount of the drug used for anesthesia maintenance, which reflected its 4–5-fold-higher potency than propofol [29]. Pain at injection was significantly lower in the ciprofol group as well as the incidence of intraoperative hypotension episodes with no difference in the postoperative kidney function recovery index such as urea, creatininemia, cystatin C, glomerular filtration rate, and urine volume; furthermore, no differences between the ciprofol and propofol groups were recorded in terms of postoperative cognitive impairment, intraoperative awareness, agitation during awakening, and postoperative nausea and vomiting [29].

Another important setting where intravenous anesthetics are used is the intensive care unit: propofol, in fact, is commonly used for the sedation of patients undergoing mechanical ventilation. Liu Y et al. investigated the potential of ciprofol to sedate patients requiring tracheal intubation in the ICU: they found that a loading dose of ciprofol 0.1–0.2 mg/kg and 0.5–1.0 mg/kg propofol followed by a continuous infusion of 0.06–0.8 mg/kg/h and 0.3–4.0 mg/kg/h, respectively, resulted in a comparable time to sedation compliance; remarkably, time from drug withdrawal to endotracheal extubation and time to full alertness were not significantly different in the ciprofol and propofol groups after a comparable duration of drug administration [30]. The incidence of adverse effects was not statistically different in the patients treated with ciprofol and propofol and in both groups no alterations in liver, renal, and coagulation function or in blood routine were recorded [30].

Nie determined the ED50 of ciprofol in children, for induction of anesthesia in a study on 36 patients: the result, with 95% CI, was 0.618 mg/kg [31]. The same study also evaluated the ED90 (with 95% CI) with the result of 0.708 mg/kg [31]. None of the patients complained of pain at the injection site. As with propofol, ciprofol in this study required a higher dosage in children than in adults.

Pei found, in a study conducted in patients undergoing adenotonsillectomy, that the optimal dosage of ciprofol for induction of anesthesia (in combination with rocuronium) is 0.6 mg/kg [32].

## 5. Intensive Care Unit (ICU)

Sedation in the intensive care unit (ICU) represents a cornerstone in the management of patients undergoing mechanical ventilation [33,34]. The main objectives include the management of agitation and pain, the reduction in metabolic stress, and the optimization of patient–ventilator interaction [35,36]. However, the balance between depth of sedation, hemodynamic stability, and pharmacological safety remains complex and of great clinical relevance [37,38]. International guidelines recommend “light” sedation for critically ill patients, using standardized monitoring tools such as the Richmond Agitation–Sedation Scale (RASS) to assess the level of sedation [34,39,40,41]. Traditionally, the most commonly used drugs for sedation in the ICU have been benzodiazepines, propofol, and dexmedetomidine. Among these, propofol has become the reference agent due to its rapid onset, predictable metabolism, and the ability to achieve a titratable and rapidly reversible sedation [30,42]. However, its use is burdened by dose-dependent adverse effects, including hypotension, bradycardia, and hypertriglyceridemia, and in more severe cases propofol infusion syndrome, a potentially fatal complication characterized by metabolic acidosis, rhabdomyolysis, and multiorgan failure [24,43]. In recent years, a series of randomized, controlled, and prospective clinical studies have evaluated the efficacy and safety of ciprofol in ICU patients undergoing mechanical ventilation. The present review aims to critically synthesize the available evidence by comparing ciprofol and propofol in terms of sedative efficacy, hemodynamic profile, safety, and potential clinical implications.

In both animal and human models, ciprofol demonstrates rapid-distribution pharmacokinetics and hepatic metabolism with high clearance, similar to that of propofol but with a lower volume of distribution and lower effective plasma concentrations [30]. This translates into a rapid and titratable clinical effect, with a reduced incidence of hypotension and bradycardia even at equivalent doses [44,45]. In a study of children who underwent radiofrequency ablation for supraventricular tachycardia, it was demonstrated that the arrhythmia-inducing ability of ciprofol was comparable to that of propofol, confirming its hemodynamic stability [46].

The pharmacokinetic analysis conducted on twelve clinical studies (including two ICU trials) showed that the behavior of ciprofol is well described by a linear three-compartment model with first-order elimination. Factors such as age, sex, and body weight marginally influence the pharmacokinetic parameters, suggesting that no dosage adjustments are required [45]. Moreover, the exposure–safety analysis showed no significant correlation between plasma drug concentration and the incidence of hypotension, confirming its hemodynamic stability [47]. The first clinical trial that evaluated ciprofol in the intensive care setting was published by Liu et al. [47]. It was a multicenter, open-label, randomized, propofol-controlled study that involved 39 adult patients undergoing mechanical ventilation. Ciprofol was administered with a loading dose of 0.1–0.2 mg/kg and maintenance between 0.06 and 0.80 mg/kg/h, while propofol was infused at standard dosages (0.5–1.0 mg/kg loading; 0.3–4.0 mg/kg/h maintenance). Both groups successfully achieved the target sedation level (RASS −2/+1) in 100% of cases, demonstrating the clinical non-inferiority of ciprofol. However, the drug showed a trend toward a lower incidence of hypotension (7.7% vs. 23.1%) and bradycardia (3.8% vs. 7.7%), although without statistical significance due to the small sample size. Plasma concentrations of the two drugs were similar, suggesting predictable and linear pharmacokinetics.

The phase III trial conducted by Liu et al. [42] expanded the population to 135 patients hospitalized in 21 Chinese centers. The study, with a multicenter, randomized, single-blind, non-inferiority design, compared ciprofol (n = 90) and propofol (n = 45) administered at 0.3 mg/kg/h and 1.5 mg/kg/h, respectively, with a target RASS between +1 and −2. The success rate of sedation was 97.7% in the ciprofol group and 97.8% in the propofol group, confirming the non-inferiority of the new agent. Treatment-related adverse events did not differ significantly, but the recovery time after sedation was longer in the ciprofol group (*p* = 0.003), likely due to the higher potency of the drug. No cases of hypertriglyceridemia or metabolic toxicity were reported. This study provided the basis for the clinical approval of ciprofol as an ICU sedative in China in 2022, defining its standard therapeutic dosage (0.1 mg/kg bolus, 0.3 mg/kg/h infusion). In 2024, Liu et al. published a post hoc combined analysis of data from phase II and III trials [47]. The objective was to evaluate early sedation (within 30 min) and the incidence of hypotension in ventilated patients. The analysis included 174 subjects (116 ciprofol, 58 propofol) and demonstrated that the percentage of hypotension-free patients was significantly higher in the ciprofol group (93% vs. 81%; *p* = 0.018). In subgroups aged <65 years and male sex, the hemodynamic advantage was more pronounced. Furthermore, patients treated with ciprofol required fewer dose adjustments and fewer vasoactive drug interventions, suggesting a more stable and predictable pharmacodynamic profile. One of the initial limitations of the evidence regarding ciprofol concerned the duration of sedation (<24 h). To address this gap, Sun et al. (2023) developed a prospective randomized study protocol for long-term sedation (>24 h) in 112 mechanically ventilated patients [19]. The study, double-blind and propofol-controlled, had as its primary endpoint the time spent in the target sedation range. Preliminary results indicate that ciprofol maintains non-inferior efficacy and safety to propofol even during prolonged infusions, with no evidence of hepatic, renal, or metabolic toxicity. The reduced incidence of blood pressure fluctuations and the stability of hemodynamic parameters suggest a potential role of the drug in continuous sedation of critically ill patients. In the same context, Zhao et al. conducted a randomized trial on 60 septic, mechanically ventilated patients, comparing ciprofol and propofol for sedations ≥ 24 h [48]. Ciprofol showed a longer time within target sedation (72.2% vs. 22.6%) and greater sedation success (53.6% vs. 14.3%), without differences in mortality or length of stay. These data support the use of ciprofol even in patients with sepsis, where hemodynamic stability is crucial. A prospective study conducted by He et al. analyzed 548 ICU patients undergoing sedation with ciprofol or propofol and concurrent vasopressor therapy [44]. After propensity score matching correction, ciprofol significantly reduced norepinephrine requirements (0.10 vs. 0.19 μg/kg/min; *p* = 0.006), while maintaining an equivalent sedation success rate (97.1% vs. 97.4%). This study provides quantitative evidence of the hemodynamic advantage of ciprofol, suggesting that reduced systemic vasodilation and a lower impact on sympathetic tone may translate into decreased pharmacological pressor support. All included studies converge in demonstrating that ciprofol is non-inferior to propofol in achieving and maintaining target sedation levels in mechanically ventilated patients. The sedation success rate consistently exceeds 95% across all trials, regardless of the infusion duration [30,45]. The characteristics of ciprofol make it less unpredictable, with less interindividual variability, and fewer requirements for dose adjustment [47]. All of this makes ciprofol the ideal sedative for sedation protocols, with greater consistency of treatment for patients. The most important aspect, however, is its greater hemodynamic stability compared to propofol. This aspect is crucial in the ICU, especially for patients in shock who still require sedation. Greater hemodynamic stability translates into a reduced requirement for vasopressor drugs, and thus a reduction in amine-related side effects [44,45,47].

In a study on children undergoing cardiac surgery, Left Ventricular Myocardial Strain and myocardial work were evaluated: ciprofol was found to be non-inferior to propofol [49].

In addition, ciprofol, compared to propofol, has fewer adverse events in terms of PRIS (Propofol Infusion Syndrome)-like syndrome, hypertriglyceridemia, and hepatic or renal toxicity. The lipid emulsion of ciprofol contains a lower lipid concentration than that of propofol, reducing the risk of accumulation and metabolic alterations [19,45]. Moreover, no significant differences were observed regarding bradycardia or cardiac events, and the drug was well tolerated even during prolonged infusions (>24 h). Despite promising results, the available evidence presents certain limitations: all studies were conducted in Chinese populations, which may limit generalizability to Western contexts; the sample size is limited in several trials (especially phase II and single-center studies); sedation durations rarely exceed 72 h, leaving long-term effects unexplored; and there is a lack of large global multicenter studies assessing hard clinical outcomes such as mortality, duration of ventilation, and incidence of delirium. The development prospects for ciprofol in intensive care are broad. Evidence suggests that the drug may become a valid alternative or replacement for propofol, especially in patients with hemodynamic instability or at risk of metabolic complications. Future studies should focus on prolonged sedation (>72 h) to define cumulative safety and steady-state pharmacokinetics; heterogeneous populations (elderly, sepsis, renal or hepatic failure); global clinical endpoints such as extubation time, delirium incidence, and ICU mortality; and pharmacoeconomic analyses to quantify its impact on reducing indirect costs related to vasopressor use and cardiovascular complications.

Current clinical evidence supports ciprofol as an effective and safe sedative agent for ICU patients undergoing mechanical ventilation. Compared to propofol, it ensures equivalent sedative efficacy, better hemodynamic stability, and lower incidence of cardiovascular and metabolic adverse events, without the need for dosage adjustments. Although confirmation in larger international studies is still required, ciprofol emerges as a potential new standard for ICU sedation, representing a step forward toward safer, titratable, and physiologically sustainable sedation.

## 6. Total Intravenous Anesthesia (TIVA)

Total Intravenous Anesthesia represents a consolidated modality of general anesthesia based exclusively on the use of hypnotic and analgesic agents administered intravenously. Since the 1990s, propofol has constituted the cornerstone of this technique thanks to its rapid onset of action, short duration, and predictable recovery [50]. However, its cardiovascular and respiratory side effects, together with the frequent pain on injection and the risk of propofol infusion syndrome, have motivated the search for alternative molecules with an improved safety profile [51,52]. Ciprofol is a GABAA receptor agonist with higher potency and receptor affinity compared with propofol and with lower lipophilicity—features that result in faster metabolism and better hemodynamic stability [53].

**Figure 3:**
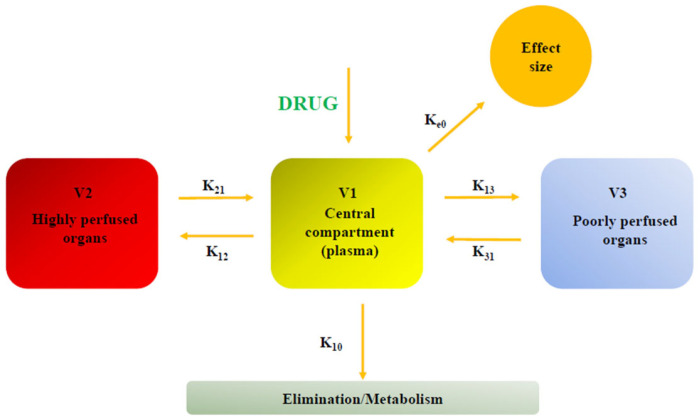
Three-compartment model of ciprofol: V1: central compartment (plasma), where drug is administered; V2: compartment constituted by highly perfused tissues (e.g., muscles), where drug is quickly distributed and which represents a temporary reservoir; V3: compartment with poor blood supply (e.g., fat), which acts as a slow releasing reservoir especially for lipophilic drugs such as ciprofol. K-constants: represent the first-order rates of drug movement (e.g., K_12_ for V1 to V2, K_21_ for V2 to V1, K_e0_ from V1 to the effect site) and elimination from V1 (K_10_).

From a pharmacokinetic point of view, ciprofol exhibits three-phase distribution (Figure 3) (t½α = 2 min, t½β = 35 min, t½γ = 6.2 h) and a predominantly non-saturable hepatic metabolism mediated by CYP2B6 and UGT1A9, with renal excretion of inactive metabolites [54].

These characteristics are associated with rapid induction and awakening, low interindividual variability, and an absence of accumulation even during prolonged infusion. Liu et al. described a pharmacokinetic/pharmacodynamic model of ciprofol analyzing the data of 334 subjects and studying the exposure–response relationship to determine pharmacodynamic parameters; they confirmed that ciprofol pharmacokinetics is properly described by a three-compartment model with a first-order elimination from the central compartment and redistribution from the deep and shallow peripheral compartments [55]; moreover, assuming the bispectral index (BIS) to be a measure of ciprofol effect, they found that the relationship between effect-site concentration of ciprofol and response is adequately described by an inhibitory sigmoidal Emax model; they also identified an optimal maintenance dose of 0.8 mg/kg/h to start 4–5 min after the induction dose; while body weight, age, sex, blood sampling site, and type of infusion (short- or long-term) were identified as statistically significant covariates affecting different pharmacokinetic parameters (i.e., clearance, distribution volume of the three compartments, and inter-compartmental clearance between the central compartment and shallow peripheral compartment), no covariates were found to significantly influence pharmacodynamic parameters.

In a randomized, controlled, non-inferiority study involving 120 patients undergoing microvascular decompression (MVD) of the facial nerve, Zhu et al. compared ciprofol (0.4 mg/kg; maintenance 0.8 mg/kg/h) and propofol (2 mg/kg; maintenance 5 mg/kg/h) in TIVA with intraoperative neurophysiological monitoring (IONM) [56]. The mean difference in CMAP between the two groups (64.7 ± 44.1 vs. 53.4 ± 35.4 μV) fell within the non-inferiority margin (95% CI −25.78 to 3.12). In this study ciprofol caused a significantly smaller decrease in blood pressure at induction (9.9 ± 7.5 mmHg vs. 17.0 ± 9.7 mmHg; *p* < 0.001). The incidence of pain on injection was lower in the ciprofol group (3.3% vs. 31.7%; *p* < 0.001). No significant differences in awakening times, anesthesia duration, or complications were observed. Ciprofol was not inferior to propofol in terms of anesthetic efficacy and neurophysiological safety, but it was superior in hemodynamic stability.

Yang et al. conducted a randomized, double-blind, non-inferiority trial on 120 women undergoing hysteroscopic surgery under TIVA with ciprofol or propofol [57].

The primary endpoint was the QoR-15 score at 24 h, a validated indicator of postoperative recovery quality; in fact QoR-15 score at 24 h was 113.5 with ciprofol and 112.5 with propofol, with a median difference of −1.0 (95% CI −3.0 to 2.0), confirming non-inferiority. During induction and maintenance, the ciprofol group showed higher MAP and HR values, indicating better cardiovascular stability. Other outcomes were as follows: no difference in time to loss or recovery of consciousness; pain on injection: 0% with ciprofol vs. 33% with propofol (*p* < 0.001); respiratory adverse events, nausea, and vomiting were similar between groups.

Ciprofol provides a postoperative recovery quality equivalent to propofol, with markedly better hemodynamic and comfort profiles.

Both drugs show similar respiratory and neurological safety profiles, but ciprofol exhibits a lower incidence of hypotension, bradycardia, and injection pain [44,56,57].

No cases of intraoperative movement, awareness, or serious complications were reported. The reduction in intraoperative norepinephrine consumption with ciprofol suggests less sympathetic depression, a relevant aspect in frail or elderly patients.

Wang verified the ED50 of ciprofol in combination with fentanyl for laryngeal mask airway insertion in a study in children [58]. The results of this study demonstrated that both the 2 mcg/kg fentanyl and 1 mcg/kg fentanyl doses reduced the ED50 of ciprofol: in the ciprofol + fentanyl 1 mcg/kg association the ED50 was 0.67 mg/kg while in the ciprofol + fentanyl 2 mcg/kg association the ED50 was 0.48 mg/kg [58].

The results of the main clinical trials confirm that ciprofol is not inferior to propofol in terms of anesthetic efficacy in TIVA. The main advantage of ciprofol lies in its cardiovascular stability and better local tolerability, aspects attributable to its higher receptor affinity and lower free plasma concentration.

From a clinical standpoint, these results are particularly relevant in high hemodynamic risk settings—such as functional neurosurgery or ambulatory surgery—where blood pressure fluctuations and vasopressor consumption represent an operative limitation.

However, the available literature still presents limitations: sample sizes are small, follow-ups are short, and the studied populations are selected (ASA I–III). Multicenter studies on long-duration procedures and critically ill patients are required to fully define the metabolic and neurological safety of ciprofol. In addition, data on pediatric pharmacodynamics and compatibility with other agents in multimodal TIVA protocols are still lacking.

Given its greater pharmacodynamic predictability, ciprofol can be a versatile anesthetic for surgery. Patients with hemodynamic instability or those requiring intraoperative neurophysiological monitoring would particularly benefit from it.

## 7. Postoperative Delirium (POD)

Postoperative Delirium (POD) is defined as an acute central nervous system syndrome characterized by fluctuations in the level of consciousness, disorientation, memory deficits, perceptual alterations, and cognitive disorganization [59,60]. Postoperative delirium has been reported to represent the second most common perioperative organ injury, with a mortality rate of approximately 6.6% when it occurs in isolation [61,62]. However, when it occurs in conjunction with other organ injuries, the mortality rate rises to 21.6% [63].

Several factors contribute to the onset of POD: advanced age, prolonged surgical duration, hypoxemia, hypotension, inflammation, stress, and the type of anesthetic agents used [64,65].

Propofol, which is widely used for the induction and maintenance of anesthesia, may be associated with hypotension, which is a risk factor for reduced cerebral perfusion [66,67,68,69,70].

Preclinical studies of ciprofol indicate a lower depressive effect on the cardiovascular system and a potential neuroprotective effect, mediated by the reduction in the inflammatory response and neuronal oxidative stress [71,72].

Several randomized, controlled trials have evaluated the effect of ciprofol on POD in comparison with propofol in different surgical settings. Liu et al. conducted a prospective, randomized, double-blind, controlled study at Liaoning Cancer Hospital on 84 elderly patients undergoing a single lobectomy through video-assisted thoracic surgery [71]. The incidence of POD was lower in the ciprofol group (7.1%) compared with the propofol group (16.7%), although the difference was not statistically significant. However, the ciprofol group showed better hemodynamic stability, with higher intraoperative MAP and SctO2 values, suggesting improved cerebral perfusion.

A subsequent study by Lu et al. [73] on 138 patients undergoing cardiac surgery with cardiopulmonary bypass revealed that the incidence of postoperative delirium in the ciprofol group was statistically lower than in the propofol group (26.69% vs. 52.31%). Hemodynamic stability and the lower incidence of intraoperative hypotension appear to contribute to this benefit, whereas no relevant differences were observed in long-term cognitive dysfunction.

Chen et al. [74] evaluated 114 elderly patients undergoing elective total hip arthroplasty, observing a lower incidence of POD in the ciprofol group (5.5%) compared with the propofol group (20%). Moreover, serum levels of the SIRT3 protein, associated with mitochondrial function and neuroprotection, were higher in patients treated with ciprofol, suggesting a possible biological mechanism in reducing the risk of delirium. However, some criticisms can be made of this study: regarding the control, the predictive value of SIRT3, and the stratification of delirium [75].

From the literature currently available, it is possible to affirm that the use of ciprofol is less burdened by emergence delirium, compared to propofol [76,77].

The possible mechanisms include greater hemodynamic stability, less impact on neuronal oxidative metabolism with preservation of mitochondrial function, and a reduction in inflammatory response and oxidative stress. However, the available literature needs to be strengthened, as the studies conducted to date have a limited simple size. Moreover, the clinical experience with propofol is long-standing, and a large body of literature, some of which is of the highest quality, is available.

## 8. Non-Operating Room Anesthesia (NORA)

In recent decades, there has been a marked increase in the number of diagnostic and therapeutic invasive procedures performed in pediatric patients outside the operating room—a field known as Non-Operating Room Anesthesia (NORA) [78]. Clinicians who are less familiar with this setting often underestimate the higher risks associated with procedural sedation, which may lead to the administration of inadequate sedation or analgesia [79,80]. The unique anatomical and physiological characteristics of infants and children, combined with the unfamiliar and unconventional NORA environment, present significant challenges for anesthetic management. Inadequate sedation may result in procedural failure and have adverse psychological and physical consequences for both the patient and their family.

The depth of sedation required and the drugs to be used depend on several factors: the type of procedure, expected level of pain, the degree of movement allowed, procedural duration, patient comorbidities, and urgency. Therefore, clinicians performing sedation must be appropriately trained in drug administration, patient monitoring, sedation assessment, and complication management [81,82].

As the gold standard for diagnosing gastrointestinal diseases, digestive endoscopy has gained considerable clinical attention. Gastroscopy, in particular, is a widely used diagnostic tool for the screening and early detection of upper gastrointestinal disorders. However, due to discomfort, nausea, and pain, most patients prefer to undergo the procedure under sedation. Despite its advantages, painless gastroscopy is not without drawbacks: excessive sedation may delay recovery, prolong hospital stay, increase procedural costs, and elevate the risk of respiratory and cardiovascular complications [83].

The introduction of sedative agents and the development of comfortable anesthesia techniques during endoscopy have helped reduce patient anxiety and pain. Propofol remains one of the most commonly used intravenous anesthetics for both anesthesia and procedural sedation. Nevertheless, in clinical practice, propofol has several limitations, including a narrow therapeutic window, dose-dependent cardiovascular and respiratory depression, and a high incidence of pain at the injection site [84]. Consequently, there is a need to develop alternative anesthetic agents that offer better efficacy and safety profiles.

Notably, recent studies have shown that the incidence of injection pain with ciprofol is markedly lower than that observed with propofol, ranging between 4.9% and 16.7%. Propofol, a phenol derivative, activates the kallikrein–kinin system, leading to bradykinin release, vasodilation, and increased vascular permeability, which irritate the skin, mucosa, and vascular endothelium [85]. In contrast, ciprofol contains a cyclopropyl group that reduces the molecule’s lipophilicity and enhances its affinity for GABA-A receptors. As a result, it produces equivalent sedative effects at lower doses while reducing injection pain [6,23].

Lidocaine, known for its local anesthetic and antiarrhythmic properties, can reduce pain perception, inflammation, and anesthetic consumption when administered intravenously. The combination of ciprofol (ED50 ≈ 1.22) and lidocaine (1.5 mg/kg bolus + 1.5 mg/kg/h infusion) significantly decreases the target concentration of ciprofol required for sedation [86].

The concomitant use of intravenous anesthetics and low-dose analgesics has been shown to provide substantial benefits in endoscopic anesthesia. In particular, combining a hypnotic agent (such as ciprofol) with a short-acting opioid (such as remifentanil or alfentanil) enhances sedation quality during gastroscopy. Remifentanil is an ultra-short-acting opioid that is rapidly metabolized to an inactive form. Unlike other synthetic opioids that undergo hepatic metabolism, remifentanil contains an ester linkage that undergoes rapid hydrolysis by non-specific tissue and plasma esterases. Thus, it does not accumulate in the body, maintaining a constant half-life of approximately four minutes even after prolonged infusion. The combination of ciprofol and remifentanil is considered a safe and effective strategy for sedation during endoscopy in school-aged children. The ED50 of ciprofol decreases from 1.32 µg/mL (with 1 ng/mL of remifentanil) to 0.92 µg/mL (with 3 ng/mL of remifentanil) [87].

Alfentanil is a potent synthetic μ-opioid receptor agonist with a short duration of action. It is a derivative of fentanyl, possessing approximately one-tenth of its potency and one-third of its duration, but with an onset five times faster. Chan et al. identified 7 µg/kg of alfentanil, when combined with an intravenous anesthetic, as the most effective analgesic regimen for painless gastroscopy [88].

Current evidence suggests that ciprofol may represent a significant advancement in modern intravenous sedation, providing an optimal balance of efficacy, safety, and patient comfort. Yang et al. demonstrated that while propofol has a shorter induction time in conventional surgical settings, ciprofol achieves faster induction in non-operating room environments [89]. Similarly, Zhong et al. found no significant difference in induction time between the two agents, indicating that ciprofol provides a rapid and predictable onset of sedation comparable to propofol [48].

Statistically significant findings indicate that propofol allows a slightly shorter recovery time compared with ciprofol [89]. However, the analysis by Zhong et al. revealed no significant difference between the two drugs [48].

In addition ciprofol is associated with a lower incidence of respiratory depression; episodes of hypoxia were rare, transient, and easily resolved with tactile stimulation or increased oxygen flow [48,89].

Drug-related bradycardia appears to be more frequent with ciprofol during surgical procedures, whereas in non-surgical settings its incidence is lower, probably related to the anesthesia plan [89].

Nie compared the association between propofol–remifentanil versus ciprofol–remifentanil in pediatric patients undergoing fibroscopy [90]. In this study, the ciprofol–remifentanil combination demonstrated a lower incidence of hypotension and desaturation, and thus a better safety profile [90].

In conclusion, ciprofol has proven to be an effective and safe sedative for NORA procedures, with no clinically significant adverse reactions reported. However, given its recent introduction, clinical experience and research on ciprofol remain relatively limited.

## 9. Discussion

The search for a “perfect” hypnotic induction has always been a hot topic in anesthesia research, especially in pediatric anesthesia [91]. The need for a safe, effective, and well-tolerated drug has always been a priority for anesthesia. Inducing patients, even in critical conditions, or sedating them in the ICU setting, requires a general anesthetic with tolerable hemodynamic consequences [92]. Pediatric patients are unique due to their anatomical and physiological characteristics, which often, if not always, do not correspond to drugs specifically designed for them [93]. Conducting research in pediatrics is difficult and expensive. Therefore, pediatric anesthesiologists are almost always forced, with minor deviations based on local regulations, to use drugs off-label [94]. Propofol is now widely used and has become a fully fledged part of clinical practice [95,96,97]. However, it has some characteristics that can be improved. In this sense, ciprofol, due to its characteristics, may represent a more advanced version of the drug, in an improved sense (Table 1). First of all, the absence of pain and discomfort at the injection site during induction represents not only an advantage for the patient, but also for the parents. Indeed, it is common practice to accompany pediatric patients to the operating room until induction, and often the parent reacts negatively to the unpleasant sensation of propofol injection. This risks becoming an unpleasant memory in the parents’ minds. Furthermore, it should be remembered that some patients must undergo repeated sedation over the course of days and may not have central vascular access. Beyond patient comfort, the impact of the hypnotic induction on hemodynamics is obviously also important, and in this sense, ciprofol appears safer than propofol, which sometimes has significant consequences [98,99].

## 10. Conclusions

Although scientific evidence in pediatrics is still limited, the literature on ciprofol in adult patients is beginning to be robust and of good quality. Ciprofol’s significant advantages may be an advantage in pediatric anesthesia.

## Figures and Tables

**Table 1:** Clinical evidence on ciprofol use in pediatric anesthesia.

Domain	Key Findings	Evidence [Ref.]
**Anesthesia induction dose**	Effective pediatric induction achieved (ED50 0.62 mg/kg; ED90 0.71 mg/kg).	Nie et al. [31]
**Laryngeal mask airway insertion**	Fentanyl reduced ciprofol dose required for smooth laryngeal mask airway insertion.	Wang et al. [58]
**Endotracheal intubation**	Ciprofol 0.6 mg/kg with low dose rocuronium ensured acceptable intubation conditions.	Pei et al. [32]
**Fiberoptic bronchoscopy sedation**	Sedation efficacy comparable to propofol, with fewer hypotension and hypoxemia episodes.	Nie et al. [90]
**Pediatric cardiac surgery**	Myocardial strain and myocardial work comparable to propofol.	Qin et al. [49]
**Supraventricular tachycardia ablation**	Arrhythmia inducibility like propofol and remimazolam.	Zhang et al. [46]
**Adenoidectomy/adenotonsillectomy recovery**	Lower pediatric anesthesia emergence delirium scores during early recovery	Zeng et al. [77]

## Data Availability

Not applicable.
